# Tuberculosis of Finger Presenting As Non-healing Ulcer of Digit: A Case Report

**DOI:** 10.7759/cureus.29426

**Published:** 2022-09-21

**Authors:** Amrit Dhar, Umar H Khan, Afshan Shabir, Suhail Mantoo, Nazia Mehfooz

**Affiliations:** 1 Internal and Pulmonary Medicine, Sher-i-Kashmir Institute of Medical Sciences, Srinagar, IND; 2 Internal Medicine, Sher-i-Kashmir Institute of Medical Sciences, Srinagar, IND; 3 Geriatric Medicine, Sher-i-Kashmir Institute of Medical Sciences, Srinagar, IND

**Keywords:** histopathology, direct inoculation, dermatology, non-healing ulcer, cutaneous tuberculosis

## Abstract

Tuberculosis (TB) is a chronic granulomatous infection which most often localises to the respiratory system. Extra-pulmonary tuberculosis is prevalent in immunocompromised individuals, of which cutaneous tuberculosis is exceedingly rare (0.5-2%). Cutaneous TB presents with varied clinical morphologies, either acquired exogenously via direct inoculation on the skin or endogenously due to systemic dissemination. Diagnosis is particularly challenging due to the multitude of differential diagnoses of skin lesions. Microbiological evidence from biopsy and histopathological findings suggestive of granulomatous inflammation are needed to make a definitive diagnosis. Herein we present a rare case of tuberculosis of the finger in a middle-aged man who presented with an ulcerating and erythematous lesion. As cutaneous TB is usually misdiagnosed at the earlier stages, dermatologists and primary care physicians should keep high suspicion for cutaneous TB in any non-healing ulcers which are otherwise unexplained.

## Introduction

Tuberculosis (TB), a chronic granulomatous infection caused by *Mycobacterium tuberculosis*, is most commonly found in the lungs. However, approximately 20% of cases present with extra-pulmonary TB (EPTB). Lymphatic disease is the most common extra-pulmonary site (30%), followed by pleural, genitourinary, bone/joint, central nervous system, and peritoneal [1]. Only about 0.5-2% of EPTB cases have cutaneous TB [2]. Despite the high prevalence of tuberculosis in countries such as India, China, Indonesia, and other Southeast Asian countries, cutaneous tuberculosis is extremely rare [3]. We present a unique case of a male with a non-healing tubercular ulcer on a finger.

## Case presentation

A 45-year-old male, manual labourer, smoker, with no underlying co-morbid illness, presented with a two-month history of an ulcer on the right middle finger. It began with a minor trauma on his middle finger around the proximal interphalangeal joint on the dorsal aspect, and the wound began to heal with scab formation over the next few days. He began to notice a shallow ulcer with a reddish base and associated excoriation of skin in the surrounding area two weeks later. The lesion was painless and did not restrict finger movement. He had no other skin lesions or rashes on his body and had never had a similar complaint before. There are no systemic symptoms such as fever, chills, night sweats, or insomnia. 

He was initially seen by a local practitioner, and antibiotic (Amoxicillin clavulanate 625 mg twice day) was prescribed for two weeks, with no discernible improvement. He had otherwise previously been healthy, with no chronic morbidity, infections, or immune disorders. There was no history of a similar complaint in the family or among acquaintances, and there was no case of active tuberculosis in the family. 

**Figure 1:**
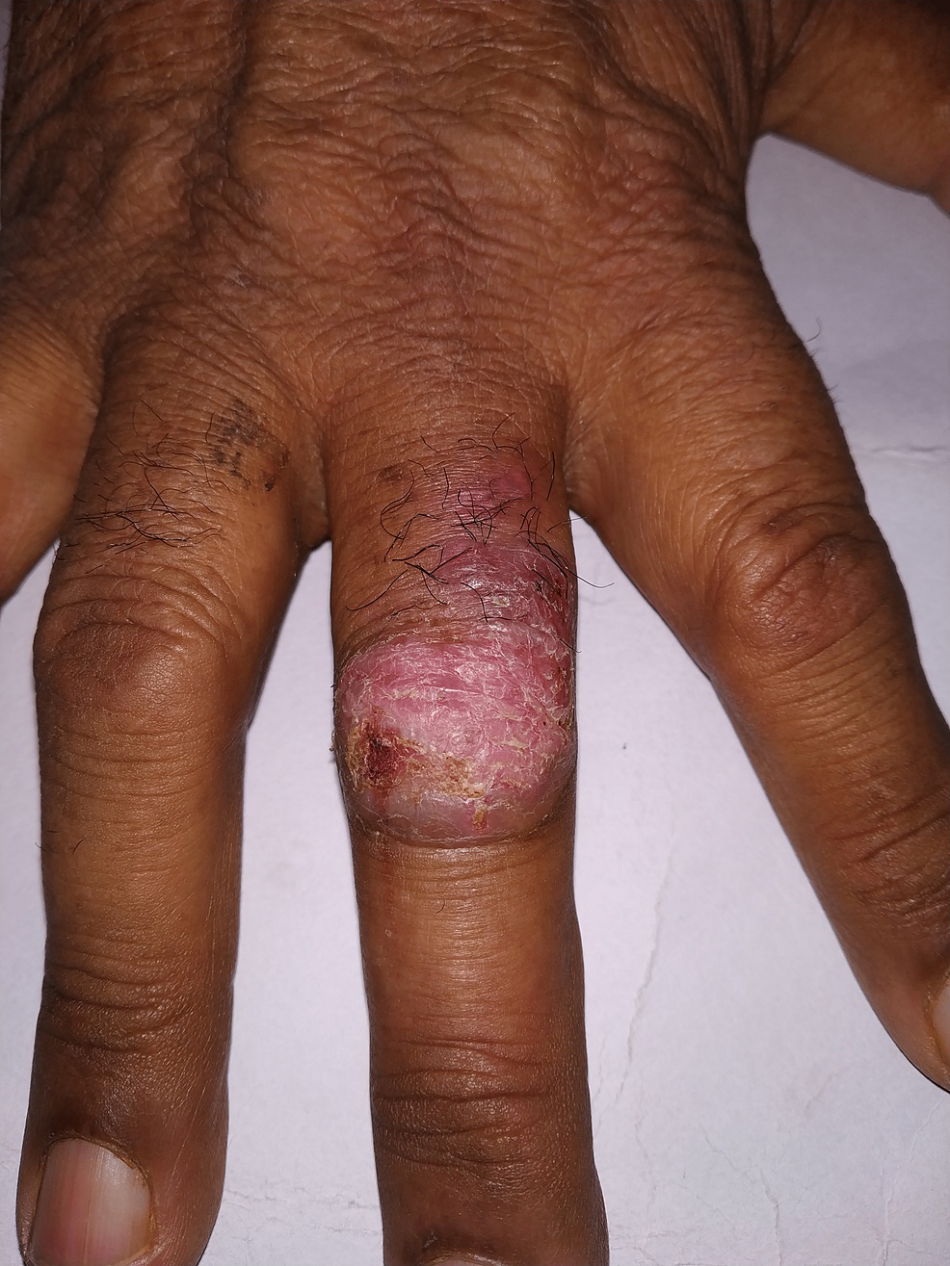
Non-healing shallow ulcer with reddish base, associated swelling, and surrounding excoriation

Local examination revealed a 3x3 cm shallow ulcer with a reddish base and mild swelling on the dorsal aspect of the proximal interphalangeal joint that extended onto the middle phalanx of the right middle finger. In the ulcer area, erythema and excoriations were observed (Figure 1). No pus could be seen when pressure was applied to the ulcer. There was no tenderness or restriction of passive or active movement of the digit. There was no sensory deficit in the area, and no regional lymphadenopathy was discovered. The skin examination of the rest of the digits was unremarkable, as was the systemic examination. 

A complete blood count revealed normocytic normochromic anaemia with haemoglobin of 10.5 g/dL (range: 13-17 g/dL for males), total leucocyte count of 6400 cells/mm3 (range: 4000-11000 cells/mm3), erythrocyte sedimentation rate of 56 mm/hr (range: 15-17 mm/hr), and C reactive protein of 24 mg/dL (range: 6 mg/dL). Renal and liver function tests were normal.

An X-ray of the finger revealed no evidence of underlying osteolysis of the middle phalanx or signs of osteomyelitis. The chest X-ray was normal, as was the finger power doppler ultrasonography. 

Following a 72-hour injection of purified protein derivative, an induration of 12 mm was observed (PPD skin test). HIV serology and tumour markers were negative using an enzyme-linked immunoabsorbent assay (ELISA). Ultrasonography for routine screening did not reveal any signs of underlying malignancy.

**Figure 2:**
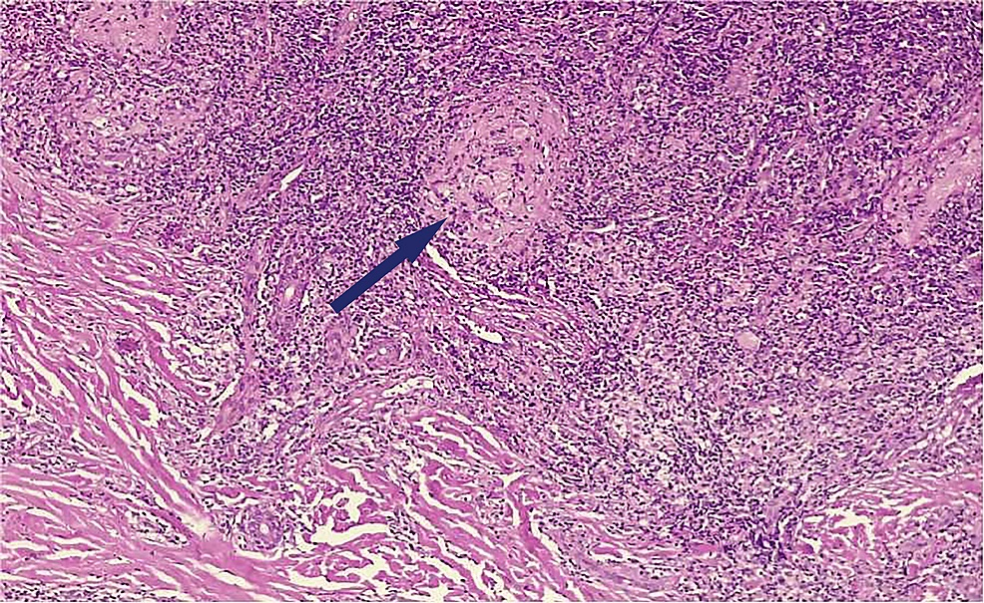
Histology section showing dense chronic inflammatory infiltrate, giant cells (Langhan type) and epithelial cell granulomas with caseating necrosis (arrow)

A skin biopsy from the edge of the ulcer was taken and sent for histopathological examination (HPE). The biopsy sample was also sent for culture, smear for acid-fast bacilli (AFB), gram stain, fungal stain, and tissue for TB-PCR (TB-polymerase chain reaction). Tissue culture, gram, and fungal staining were negative. However, the smear for AFB and tissue for TB-PCR returned positive. The histology of the lesion (Figure 2) revealed intense chronic granulomatous inflammation with caseating necrosis. 

Diagnosis of cutaneous TB via exogenous route was made. The patient was started on the first-line anti-TB regimen for six months (consisting of isoniazid, rifampicin, pyrazinamide, and ethambutol for two months followed by isoniazid and rifampicin for four months). On follow-up, after six months, the patient reported healing of the lesion.

## Discussion

Cutaneous TB is a rare form of EPTB, accounting for less than two percent of all cases [2]. The non-specific clinical features of the lesions are frequently overlooked in the differential diagnosis of erythematous and/or ulcerating skin lesions. The clinical manifestations of cutaneous tuberculosis vary, and infection can occur primarily through two routes. Exogenous or endogenous routes, each have their own clinical presentation [4,5]. In the exogenous route, cutaneous inoculation occurs directly on the skin or after a break in the skin integrity, usually following trauma, as in our case (tuberculosis chancre, tuberculosis verrucosa cutis, and some cases of lupus vulgaris) or the endogenous route, cutaneous involvement occurs secondarily, through hematogenous spread from a distant tuberculosis focus or by contiguity from an already established focus (lupus vulgaris, scrofuloderma, miliary tuberculosis, and orificial tuberculosis) [6-8]. 

Finger as a site of cutaneous tuberculosis is uncommon, with only few cases reported to date. The vast majority of cases of tuberculosis of the finger manifest as tenosynovitis or dactylitis. Another classification divides it into two classes: multi-bacillary (tuberculous chancre, scrofuloderma, tuberculosis orificialis, acute miliary tuberculosis, gummatous tuberculosis) and pauci-bacillary ( tuberculous verrocosa cutis, lupus vulgaris, tuberculids). Histological examination with Ziehl-Nielsen staining for AFB is used to differentiate between the two categories. Mycobacteria in large numbers can be easily identified in multi-bacillary forms, whereas bacilli are seen sparsely in pauci-bacillary forms. This classification has been widely compared to the description of *Mycobacterium leprae* in leprosy by Ridley and Jopling [2]. 

Cutaneous tuberculosis primarily affects immunocompromised individuals, as evidenced by the high incidence in HIV-infected subjects and patients undergoing immunosuppressive therapy [9]. Furthermore, it has been identified as a complication of immune reconstitution caused by anti-retroviral therapy [10]. Because none of these characteristics was present in our case, it is important to note that cutaneous TB can be found in healthy normal people, particularly in TB-endemic areas.

The differential diagnosis of cutaneous infections includes infections caused by non-tuberculous mycobacteria (NTM) apart from cutaneous TB [11]. NTM infections can occur as a result of a traumatic injury, surgery, or cosmetic procedure [12]. Histological analysis of biopsy material can help distinguish between the two, as PPD and AFB staining are usually positive [13]. Granulomas in cutaneous TB are most commonly found in the upper and mid dermis, with caseous necrosis and well-formed epithelioid cells containing Langerhans giant cells and lymphocytes [14]. In contrast, neutrophilic infiltration with interstitial granulomas and small vessel proliferation are observed in NTM cutaneous infections [13].

To diagnose cutaneous tuberculosis, a combination of clinical suspicion, microbiological tests, including PCR (the most sensitive), and histology must be considered. Rapid detection of *M. tuberculosis* is critical for the diagnosis of some forms of cutaneous tuberculosis, where manifestations can lead to squamous cell carcinoma or surgical amputation of the affected area. Once diagnosed, treatment is similar to that of pulmonary tuberculosis. A two-month course of rifampicin, isoniazid, pyrazinamide, and ethambutol is followed by a four-month course of only rifampicin and isoniazid. In addition to medical therapy, for skin lesions, surgical excision, cryotherapy, and electrocautery may be used for cosmetic reasons [5]. 

## Conclusions

Cutaneous tuberculosis is a rare presentation frequently misdiagnosed in its early stages. Dermatologists and primary care physicians should look for cutaneous tuberculosis in any non-healing ulcers that are otherwise unexplained. This can happen in previously healthy people with no TB exposure and systemic symptoms, making diagnosis difficult. Diagnosis entails a high level of clinical suspicion, microbiological tests, and tissue histology.

## References

[1] Rieder HL, Snider DE Jr, Cauthen GM (1990). Extrapulmonary tuberculosis in the United States. Am Rev Respir Dis.

[2] Bravo FG, Gotuzzo E (2007). Cutaneous tuberculosis. Clin Dermatol.

[3] (2022). Global Tuberculosis Report. https://apps.who.int/iris/bitstream/handle/10665/336069/9789240013131-eng.pdf.

[4] Barbagallo J, Tager P, Ingleton R, Hirsch RJ, Weinberg JM (2002). Cutaneous tuberculosis: diagnosis and treatment. Am J Clin Dermatol.

[5] Sethi A (2012). Tuberculosis and infections with atypical Mycobacteria. Fitzpatrick's Dermatology in General Medicine.

[6] De Maio F, Trecarichi EM, Visconti E, Sanguinetti M, Delogu G, Sali M (2016). Understanding cutaneous tuberculosis: two clinical cases. JMM Case Rep.

[7] Concha RM, Fich SF, Rabagliati BR, Pinto SC, Rubio LR, Navea DO, González BS (2011). Cutaneous tuberculosis: two case reports and review (Article in Spanish). Rev Chilena Infectol.

[8] Dias MF, Bernardes Filho F, Quaresma MV, Nascimento LV, Nery JA, Azulay DR (2014). Update on cutaneous tuberculosis. An Bras Dermatol.

[9] Handog EB, Gabriel TG, Pineda RT (2008). Management of cutaneous tuberculosis. Dermatol Ther.

[10] Huiras E, Preda V, Maurer T, Whitfeld M (2008). Cutaneous manifestations of immune reconstitution inflammatory syndrome. Curr Opin HIV AIDS.

[11] Mitha M, Naicker P, Taljaard J (2011). Cutaneous Mycobacterium kansasii infection in a patient with AIDS post initiation of antiretroviral therapy. J Infect Dev Ctries.

[12] Bhambri S, Bhambri A, Del Rosso JQ (2009). Atypical mycobacterial cutaneous infections. Dermatol Clin.

[13] Min KW, Ko JY, Park CK (2012). Histopathological spectrum of cutaneous tuberculosis and non-tuberculous mycobacterial infections. J Cutan Pathol.

[14] Bhutto AM, Solangi A, Khaskhely NM, Arakaki H, Nonaka S (2002). Clinical and epidemiological observations of cutaneous tuberculosis in Larkana, Pakistan. Int J Dermatol.

